# Feasibility and discriminatory value of tissue motion annular displacement in sepsis-induced cardiomyopathy: a single-center retrospective observational study

**DOI:** 10.1186/s13054-022-04095-w

**Published:** 2022-07-18

**Authors:** Jieqiong Song, Yao Yao, Shilong Lin, Yizhou He, Duming Zhu, Ming Zhong

**Affiliations:** Department of Critical Care Medicine, Zhongshan Hospital, Fudan University, Shanghai, 200030 China

**Keywords:** Sepsis, Cardiomyopathy, Speckle tracking echocardiography, Tissue motion annular displacement, Mortality

## Abstract

**Background:**

There is no formal diagnostic criterion for sepsis-induced cardiomyopathy (SICM), but left ventricular ejection fraction (LVEF) < 50% was the most commonly used standard. Tissue motion annular displacement (TMAD) is a novel speckle tracking indicator to quickly assess LV longitudinal systolic function. This study aimed to evaluate the feasibility and discriminatory value of TMAD for predicting SICM, as well as prognostic value of TMAD for mortality.

**Methods:**

We conducted a single-center retrospective observational study in patients with sepsis or septic shock who underwent echocardiography examination within the first 24 h after admission. Basic clinical information and conventional echocardiographic data, including mitral annular plane systolic excursion (MAPSE), were collected. Based on speckle tracking echocardiography (STE), global longitudinal strain (GLS) and TMAD were, respectively, performed offline. The parameters acquisition rate, inter- and intra-observer reliability, time consumed for measurement were assessed for the feasibility analysis. Areas under the receiver operating characteristic curves (AUROC) values were calculated to assess the discriminatory value of TMAD/GLS/MAPSE for predicting SICM, defined as LVEF < 50%. Kaplan–Meier survival curve analysis was performed according to the cutoff values in predicting SICM. Cox proportional hazards model was performed to determine the risk factors for 28d and in-hospital mortality.

**Results:**

A total of 143 patients were enrolled in this study. Compared with LVEF, GLS or MAPSE, TMAD exhibited the highest parameter acquisition rate, intra- and inter-observer reliability. The mean time for offline analyses with TMAD was significantly shorter than that with LVEF or GLS (*p* < 0.05). According to the AUROC analysis, TMADMid presented an excellent discriminatory value for predicting SICM (AUROC > 0.9). Patients with lower TMADMid (< 9.75 mm) had significantly higher 28d and in-hospital mortality (both *p* < 0.05). The multivariate Cox proportional hazards model revealed that BMI and SOFA were the independent risk factors for 28d and in-hospital mortality in sepsis cases, but TMAD was not.

**Conclusion:**

STE-based TMAD is a novel and feasible technology with promising discriminatory value for predicting SICM with LVEF < 50%.

**Supplementary Information:**

The online version contains supplementary material available at 10.1186/s13054-022-04095-w.

## Background

Sepsis-induced cardiomyopathy (SICM), initially described in the 1980s, is generally defined as an acute and reversible cardiac dysfunction that involves decreased left and/or right ventricular systolic and/or diastolic function, left ventricular dilatation, and absence of acute coronary syndrome [1]. Although there is no formalized or consensus definition, the left ventricular ejection fraction (LVEF) of less than 50% is often considered as indicative of SICM [2–5] in most literature. SICM is commonly found in patients with sepsis, especially those with septic shock [6]. The reported incidence of SICM defined as LVEF < 50% ranges widely from 10 to 70% [7]. Notably, mortality among septic patients with SICM is 2 ~ 3 times higher than those without SICM [6, 8, 9].

Echocardiography is considered the most important method for the diagnosis of SICM. Every patient with unstable hemodynamics is suggested to receive echocardiography examination [10, 11]. In addition to LVEF, mitral annular plane systolic excursion (MAPSE) is another simple method which is obtained by M-mode echocardiography and commonly used to detect LV dysfunction. Speckle tracking echocardiography (STE) is a relatively new technology for LV strain measurement, which is characterized by angle-independence and semi-automatization [12, 13]. STE assesses the cardiac function by tracking the displacement of groups of acoustic greyscale “speckles” frame by frame through the whole myocardium [14]. According to its working principle, STE directly measures the myocardium deformation; therefore, it is less affected by the LV loading conditions and myocardial compliance. Global longitudinal strain (GLS) has demonstrated to be a more sensitive indicator of LV dysfunction in sepsis [15–17].

LVEF and GLS measurements require a clear tracing of the endocardium; thus, high-quality echocardiographic imaging is essential. However, this is challenging because patients should be placed in the left lateral decubitus position (the routine ultrasound examination position). Ideal position is difficult to achieve for critically ill patients especially those with unstable hemodynamics, restriction due to surgical drainage tubes or organ support devices, such as continuous renal replacement therapy (CRRT) and extracorporeal membrane oxygenation (ECMO). Adequate image quality may also be challenging in patients with chronic obstructive pulmonary disease (COPD) or on positive pressure ventilation.

Tissue motion annular displacement (TMAD) is a novel speckle tracking indicator that quickly assesses the LV longitudinal systolic function [18–20]. TMAD tracks the displacement of the mitral annulus and the apex of the left ventricle instead of the whole LV endocardium. Less requirement for the quality of the echocardiographic image means less time consumed to trace and adjust the outline of entire endocardium and higher acquisition rate of echocardiographic parameters [18, 21, 22]. Therefore, TMAD may be a valuable tool to evaluate cardiovascular diseases, in particular, to perform early diagnosis and assessment of therapeutic efficacy [23, 24]. However, the application of TMAD in SICM is rarely reported. Considering the above, we hypothesized that TMAD plays an important role in evaluating LV longitudinal systolic function and discriminating SICM in septic patients. This study aimed to assess the feasibility and the discriminatory value of TMAD for predicting SICM defined as LVEF < 50%, as well as the prognostic value of TMAD in septic patients.

## Methods

### Study population

A single-center retrospective observational study was conducted in Zhongshan Hospital Fudan University. Patients who were admitted to the surgical intensive care unit (SICU) from March 2019 to July 2021 and met the following criteria were enrolled as study subjects: (1) age ≥ 18 years; (2) diagnosis of sepsis or septic shock; and (3) underwent echocardiography examination within the first 24 h after admission to SICU. Those who met any of the following criteria were excluded: (1) poor echocardiographic image quality or incomplete echocardiographic data; (2) incomplete clinical data; (3) history of valvular heart disease; (4) chronic heart failure (CHF) with the history of coronary artery disease (CAD), hypertrophic cardiomyopathy (HCM) or other ischemic heart diseases; (5) holder of cardiac implanted device; (6) atrial fibrillation; (7) refusal to participate in the study; and (8) loss to follow-up and fail to get consent. This project was approved by the Ethics Committee of the Zhongshan Hospital Fudan University (Approval No: B2021-501R). All participants or legal representatives were contacted through telephone calls and signed informed consent.

### Basic characteristics and clinical information collection

Basic characteristic and clinical data from all participants were collected within the first 24 h after admission to SICU. Basic information was collected from the electronic medical record system, including age, gender, body mass index (BMI), comorbidity, medication history, septic source, the Acute Physiology and Chronic Health Evaluation II (APACHE II) score and the Sequential Organ Failure Assessment (SOFA) score. Clinical information comprised heart rate, mean arterial pressure (MAP) and central venous pressure (CVP), cardiac troponin T (cTnT), N-terminal pro brain natriuretic peptide (NT-proBNP) and creatine kinase-MB (CK-MB).

### Conventional echocardiographic data

All conventional echocardiography examinations in our study were conducted using a CX50 CompactXtreme Ultrasound System (Philips, MA, USA) with a 1–5 MHz-phased array transducer. The recorded parameters included: left atrial dimension, LV end-systolic and end-diastolic volume (LVESV/LVEDV), LVEF, velocity–time integral of LV outflow tract (LVOT VTI), cardiac output (CO), early (E) and late (A) diastolic trans-mitral inflow velocity, early (e′) and late (a′) lateral diastolic mitral annular tissue velocity, maximal lateral systolic mitral annular tissue velocity (MA Smax), mitral annular plane systolic excursion (MAPSE), tricuspid annular plane systolic excursion (TAPSE), pulmonary arterial systolic pressure (PASP). LV volumes and LVEF were calculated by the modified biplanar Simpson method. E and A velocities were measured using pulsed wave (PW) Doppler at the tip of the mitral valve, and e′ and a′ tissue velocities were measured using PW tissue Doppler in the apical four-chamber view. E/A ratio and E/e′ ratios were calculated. LVEF < 50% was considered as the diagnostic criteria of SICM [13, 14].

### Speckle tracking echocardiographic data

STE analysis was performed offline and averaged by two independent investigators who were trained by an echocardiography software engineer. During the echocardiographic analysis, the investigators were blinded to the patients’ clinical conditions. STE analysis was performed by the QLAB software, version 9.0 (Philips Healthcare, MA, USA).

#### GLS

**Fig. 1:**
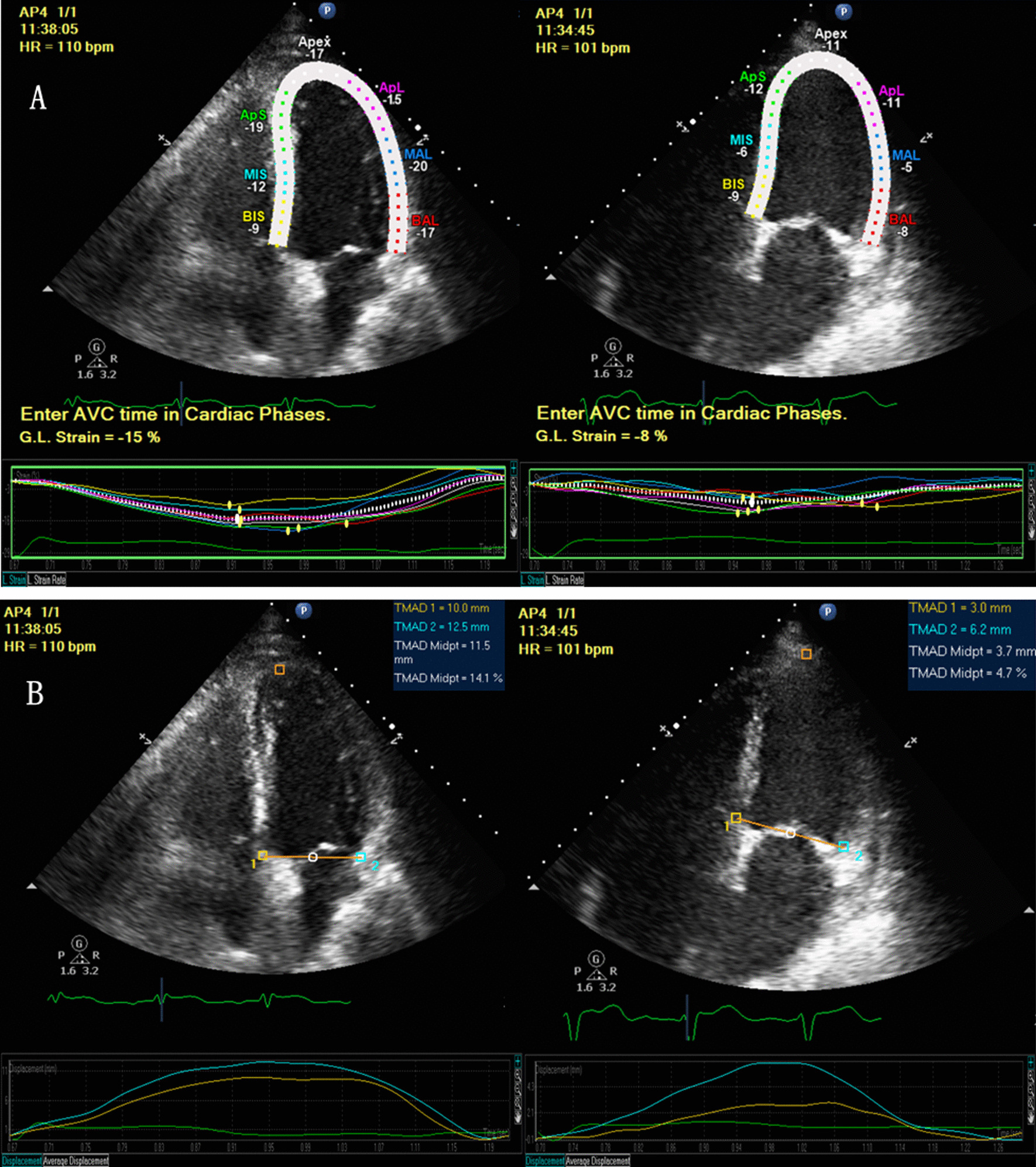
STE of septic patients. **A** Four-chamber view of the GLS measurement (left: one non-SICM patient with GLS -15%; right: one SICM patient with GLS -8%). **B** Four-chamber view of the TMAD measurement (left: one non-SICM patient with TMADMid 11.5 mm; right: one SICM patient with TMADMid 3.7 mm). BIS, basal inferior septum; MIS, mid-inferior septum; ApS, apical septum; BAL, basal anterolateral; MAL, mid-anterolateral; ApL, apical lateral; TMAD1, septal tissue motion annular displacement; TMAD2, lateral tissue motion annular displacement; TMADMid, midpoint tissue motion annular displacement; and %TMAD, the percentage value of the midpoint displacement in relation to the total length of the left ventricle

To distinguish the different phases of the cardiac cycle, the opening and closing times of the mitral and aortic valves were derived from the electrocardiograph. For GLS evaluation, the semi-automated CMQ package of the QLAB software was used along the apical longitudinal axis of the left ventricle in four-, two-, and three-chamber views. When the system focused on two mitral annular and apical points of the three left ventricle longitudinal sections, the software automatically tracked the speckles of the endocardial border throughout the cardiac cycle. Any part of the myocardium that seemed imprecisely tracked was manually and carefully modified by the investigators. In each myocardial segment, the strain was measured, and GLS was calculated by averaging the peak strain values of the whole 17 segments during systole [15] (Fig. 1A). Images with frame rate > 50 frames/s were selected for analysis.

#### TMAD

TMAD was measured offline in the apical four-chamber views using the TMAD package of the QLAB software. To assess TMAD, three regions of interest (ROIs) were selected: the septal (TMAD1) and lateral (TMAD2) areas of the mitral annulus, as well as the apex of the LV. The midpoint (TMADMid) between the two annuli ROIs was automatically detected after setting these three ROIs. Then, tracking was automatically performed frame by frame and the average of the base-to-apical displacement of the two mitral annulus ROIs was calculated in millimeters. TMADMid was also calculated in millimeters, and a percentage value of the midpoint displacement in relation to the total length of the left ventricle was calculated (%TMAD) (Fig. 1B, Additional file 1). For the TMAD technique, the only manual procedure was setting the ROIs [25]. All echocardiographic data were recorded over three consecutive cardiac cycles and then averaged.

### Feasibility analysis

Feasibility was defined as echocardiographic parameters acquisition rate, intra-observer and inter-observer variability and time consumed for measurement.

First of all, the parameter acquisition rates of LVEF, MAPSE, GLS and TMAD in this study were calculated, respectively. Secondly, to evaluate the repeatability of the speckle tracking echocardiographic data, a random sample of 20 echocardiographic examinations (LVEF, MAPSE, GLS and TMAD, respectively) was reassessed by the same investigator with a minimum interval of 4 weeks from the first evaluation as the intra-observer variability. Then, to calculate inter-observer variability, the same 20 samples were examined by another investigator who was blinded to the results of the first investigator. Intra- and inter-observer agreement was evaluated using the intraclass correlation coefficient (ICC), which ranged from + 1 (100% agreement) to − 1 (100% disagreement). ICC scores of 0.75 or higher were considered as indicators of a quality control criterion with acceptable reliability. In addition, the duration of the LVEF, MAPSE, GLS and TMAD offline analyses, respectively, was also recorded for these 20 echocardiographic examinations.

### Study outcomes

All patients were followed for 28 days after ICU admission or discharge, whichever occurred later. The primary outcomes of the study were the feasibility and discriminatory value of TMAD for SICM defined as LVEF < 50%. The secondary outcomes of the study were the feasibility and discriminatory value of MAPSE and GLS for SICM. The exploratory goal was to evaluate if TMAD, other echo or clinical variables had independent prognostic value for 28d or in-hospital mortality.

### Statistics

Continuous variables were presented as mean ± standard deviation (SD) or median and interquartile range, while categorical variables were expressed as frequency and percentage. For normally distributed continuous variables, comparisons were performed using the Student’s t-test, whereas non-normally distributed variables were analyzed with the Wilcoxon signed-rank test. Chi-squared test or Fisher’s exact test was used for categorical variables. The relationships between the selected variables were assessed by Pearson’s correlation analysis. The discriminatory values of TMAD, GLS and MAPSE for predicting SICM defined as LVEF < 50% were presented by the area under the receiver operating characteristic curves (AUROC). AUROC values were interpreted as follows: < 0.70 = poor; 0.70 to 0.80 = fair; 0.80 to 0.90 = good; > 0.90 = excellent. The optimal cutoff value of TMAD, GLS and MAPSE to discriminate SICM was determined according to the Youden index. Kaplan–Meier curves analysis with log-rank tests was performed according to the cutoff values of these echocardiographic parameters. Prognostic value of TMAD and other echo or clinical variables for 28d and in-hospital mortality were explored by Cox proportional hazard model analysis. All statistical analysis was conducted using SPSS 19.0 (IBM, Armonk, NY, USA). The graphic presentation was created by GraphPad Prism 9.0 (GraphPad Software, La Jolla, CA, USA). A *p* value < 0.05 was considered statistically significant.

## Results

### Basic characteristics and clinical information

**Fig. 2:**
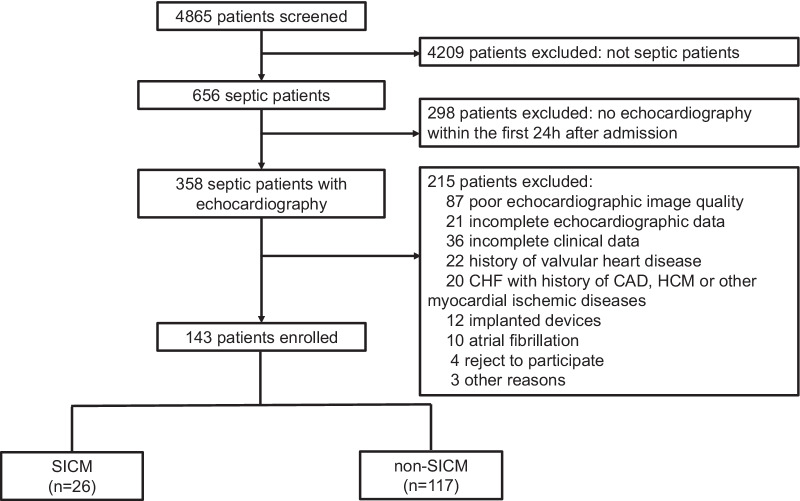
The flow diagram of patient enrollment

From March 2019 to July 2021, 4865 critically ill adult patients were consecutively admitted to SICU, and 656 of them were diagnosed with sepsis. Among these patients, 358 completed echocardiography examinations within the first 24 h after admission to SICU. Of them, 215 patients were excluded mainly for the following reasons: poor echocardiographic image quality (*n* = 87), incomplete echocardiographic data (*n* = 21), incomplete clinical data (*n* = 36), history of valvular heart disease (*n* = 22), CHF with history of CAD, HCM or other ischemic heart diseases (n = 20), holder of implanted devices (*n* = 12), atrial fibrillation (*n* = 10), refusal to participate (*n* = 4) and other reasons (*n* = 3). Finally, 143 patients were included. The flow diagram of the study is shown in Fig. 2. Of all participants, 94 patients (65.7%) were male, mean age was 69 years old, BMI was 22.9 ± 3.2, APACHE II score was 17.1 ± 6.2, and SOFA score was 6.8 ± 2.7.

According to the LVEF criteria, all patients enrolled were divided into the SICM (*n* = 26, 18.2%) or the non-SICM (*n* = 117, 81.8%) group. No significant difference was found in age, gender, BMI, comorbidity, medication history and sepsis source between the SICM and non-SICM group. However, the APACHE II score and SOFA score of the SICM group were significantly higher than that of the non-SICM group (19.9 ± 6.9 vs 16.4 ± 5.8, *p* = 0.008; 8.3 ± 3.1 vs 6.4 ± 2.5, *p* = 0.007). According to the laboratory results, patients in the SICM group had significantly higher cTnT, NT-proBNP and CK-MB within the first 24 h after admission, when compared to the non-SICM group (all *p* < 0.05). The 28d and in-hospital mortality were significantly higher in the SICM group than in the non-SICM group (34.6% vs. 13.7%, *p* = 0.011; 42.3% vs. 20.5%, *p* = 0.019, respectively). Baseline and clinical information of the patients is summarized in Table 1.

**Table 1:** Baseline characteristics and clinical information of the patients

	All patients (n = 143)		SICM (*n* = 26)		non-SICM (*n* = 117)		*p* value
Age (yrs)	69 [57–78]		73 [63–83]		68 [57–77]		0.310
Gender (male, %)	94 (65.7%)		17 (65.4%)		77 (65.8%)		0.967
BMI (kg/m^2^)	22.9 ± 3.2		22.9 ± 3.0		22.9 ± 3.3		0.949
APACHE II score	17.1 ± 6.2		19.9 ± 6.9		16.4 ± 5.8		0.008
SOFA score	6.8 ± 2.7		8.3 ± 3.1		6.4 ± 2.5		0.007
*Comorbidity* (*n*, %)							
Hypertension	52 (36.4%)		10 (38.5%)		42 (35.9%)		0.806
Diabetes mellitus	35 (24.5%)		8 (30.8%)		27 (23.1%)		0.409
COPD	18 (12.6%)		5 (19.2%)		13 (11.1%)		0.259
Peripheral vascular disease	8 (5.6%)		3 (11.5%)		5 (4.3%)		0.145
Cancer	22 (15.4%)		6 (23.1%)		16 (13.7%)		0.229
others	10 (7.0%)		6 (10.9%)		4 (4.5%)		0.184
*Medication history* (*n*, %)							
β-blocker	18 (12.6%)		5 (19.2%)		13 (11.1%)		0.259
CCB	40 (28.0%)		8 (30.8%)		32 (27.4%)		0.725
ACEI / ARB	28 (19.6%)		6 (23.1%)		22 (18.8%)		0.619
Statin	12 (8.4%)		4 (15.4%)		8 (6.8%)		0.231
*Septic source* (*n*, %)							
Abdominal	66 (46.2%)		16 (61.5%)		50 (42.7%)		0.082
Pneumonia	36 (25.2%)		9 (34.6%)		27 (23.1%)		0.220
Urinary tract	16 (11.2%)		4 (15.4%)		12 (10.3%)		0.453
Bloodstream	15 (10.5%)		4 (15.4%)		11 (9.4%)		0.368
Catheter	5 (3.5%)		1 (3.8%)		4 (3.4%)		1.0
Soft tissue	3 (2.1%)		1 (3.8%)		2 (1.7%)		1.0
Central nervous system	2 (1.4%)		1 (3.8%)		1 (0.9%)		1.0
HR (bmp)	99 ± 21		103 ± 22		96 ± 20		0.708
MAP (mmHg)	70 ± 11		64 ± 9		75 ± 11		0.126
CVP (mmHg)	9.8 ± 2.8		10.5 ± 3.1		9.3 ± 2.6		0.658
cTnT (ng/mL)	0.09 ± 0.19		0.17 ± 0.27		0.06 ± 0.07		0.002
NT-proBNP (pg/mL)	5120 ± 7687		11,910 ± 12,740		3038 ± 5147		< 0.001
CK-MB (ng/mL)	15.5 ± 43.2		10.2 ± 10.9		5.7 ± 10.3		0.006
28d mortality (*n*, %)	25 (17.5%)		9(34.6%)		16 (13.7%)		0.011
In-hospital mortality (*n*, %)	35 (24.5%)		11 (42.3%)		24 (20.5%)		0.019
Length of ICU stay (days)	7 [3–14]		10 [3–18]		6 [3–13]		0.529
Length of hospital stay (days)	19 [6, 7, 12–28]		17 [6, 7, 12–25]		19 [6, 7, 12–29]		0.091
Duration of MV (days)	37 [12–132]		41 [16–192]		37 [12–134]		0.890

BMI, body mass index; APACHE II, Acute Physiology and Chronic Health Evaluation II; SOFA, Sequential Organ Failure Assessment; COPD, chronic obstructive pulmonary disease; CCB, calcium channel blocker; ACEI, angiotensin-converting enzyme inhibitor; ARB, angiotensin receptor blocker; HR, heart rate; MAP, mean arterial pressure; CVP, central venous pressure; ICU, intensive care unit; MV, mechanical ventilation; cTnT, cardiac troponin T; NT-proBNP, N-terminal pro-B type natriuretic peptide; CK-MB, creatine kinase-MB

### Feasibility of different echocardiographic parameters

#### Acquisition rates

**Fig. 3:**
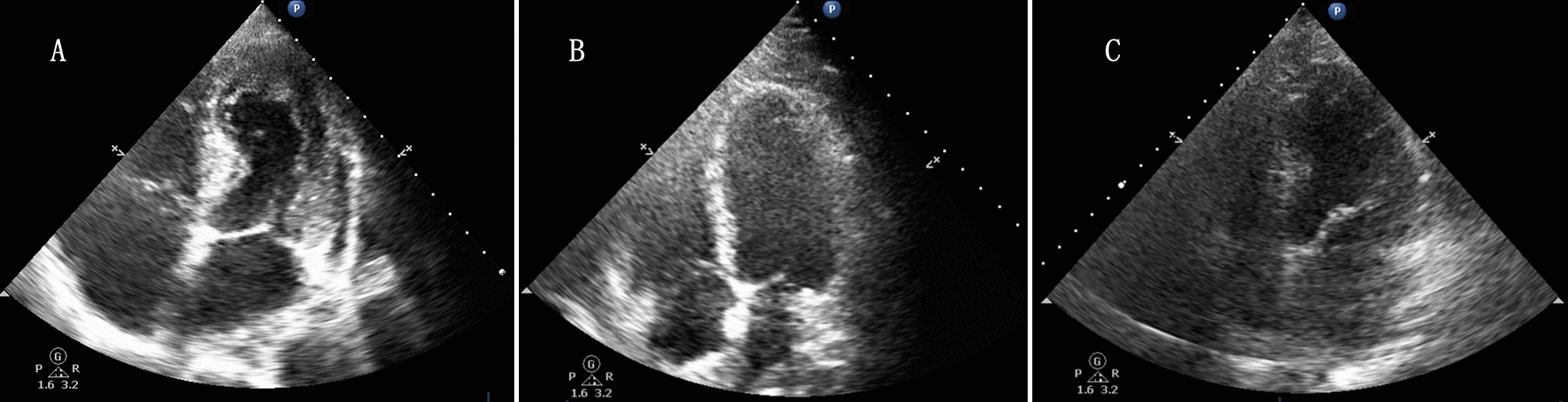
Transthoracic echocardiography of different imaging qualities performed in septic patients. **A** One patient with a legible image of the entire endocardium (LVEF, MAPSE, GLS and TMAD could be measured accurately); **B** One patient with an image that was legible for the apex and mitral annulus but indistinct for the LV lateral wall (TMAD and MAPSE could be measured, but LVEF and GLS could not be achieved accurately); **C** One patient with an indistinct image of the entire endocardium. (None of LVEF, MAPSE, GLS or TMAD could be measured accurately.) LVEF, left ventricle ejection fraction; MAPSE, mitral annular plane systolic excursion; GLS, global longitudinal strain; and TMAD, tissue motion annular displacement

Among the 87 patients with poor echocardiographic image quality, 35 patients had inadequate imaging of the entire endocardium (LVEF, MAPSE, GLS and TMAD could not be measured accurately), and the other 52 patients had adequate imaging of the apex and mitral annulus but inadequate imaging of the other endocardium parts, especially the lateral wall of the LV (MAPSE and TMAD could be measured, but LVEF and GLS could not be assessed) (Fig. 3). The acquisition rates of MAPSE and TMAD were significantly higher than that of LVEF or GLS (84.8% vs. 62.2%, *p* < 0.001).

#### Intra- and inter-observer variabilities and time-consuming for measurement

Intra- and inter-observer variabilities for LVEF, MAPSE, GLS and TMAD were assessed and are shown in Table 2. TMAD had the highest ICC value when compared to LVEF, MAPSE or GLS. The time consumed for TMAD measurement was similar to MAPSE (41 s ± 9 s vs. 36 s ± 8 s, *p* = 0.216), but significantly shorter than that for LVEF or GLS (41 s ± 9 s vs. 83 s ± 15 s, *p* < 0.001; 41 s ± 9 s vs. 70 s ± 11 s, *p* = 0.006, respectively).

**Table 2:** Intra- and inter-observer reliabilities for LVEF, GLS, MAPSE and TMAD

Intra-observer variability	ICC	95% CI	*p* value
LVEF	0.943	0.862–0.977	< 0.001
GLS	0.871	0.703–0.947	< 0.001
MAPSE	0.866	0.693–0.945	< 0.001
TMAD	0.962	0.907–0.985	< 0.001

Inter-observer variability	ICC	95% CI	*p* value
LVEF	0.912	0.791–0.964	< 0.001
GLS	0.896	0.758–0.958	< 0.001
MAPSE	0.831	0.622–0.930	< 0.001
TMAD	0.965	0.914–0.986	< 0.001

ICC, intraclass correlation coefficient; LVEF, left ventricle ejection fraction; GLS, global longitudinal strain; MAPSE, mitral annular plane systolic excursion; TMAD, tissue motion annular displacement

#### Echocardiographic data of SICM and non-SICM patients

Conventional and speckle tracking echocardiographic data of all patients are shown in Table 3. LVEF, MAPSE, MA Smax, LVOT VTI, CO, TAPSE, TMAD 1, TMAD 2, TMADMid and %TMAD were significantly lower in the SICM group than in the non-SICM group (all *p* < 0.05). Meanwhile, LVESV and GLS in those with SICM were significantly higher than in non-SICM patients (all *p* < 0.05).

**Table 3:** Echocardiography data of the patients

	All patients (*n* = 143)	SICM (*n* = 26)	Non-SICM (*n* = 117)	*p* value
LA (mm)	38.5 ± 3.4	29.9 ± 12.6	34.7 ± 4.4	0.353
LVESV (mL)	35.6 ± 14.0	48.4 ± 13.8	32.7 ± 12.4	< 0.001
LVEDV (mL)	82.9 ± 27.0	82.5 ± 20.1	83.1 ± 28.4	0.894
LVEF (%)	57.1 ± 9.3	41.7 ± 6.7	60.6 ± 5.5	< 0.001
MAPSE (mm)	12.6 ± 3.9	9.4 ± 2.6	13.4 ± 3.6	< 0.001
MA Smax (cm/s)	11.8 ± 3.1	9.5 ± 2.4	12.4 ± 3.0	< 0.001
LVOT VTI (cm)	19.7 ± 5.5	14.3 ± 3.6	21.1 ± 5.0	< 0.001
CO (L/min)	5.1 ± 1.7	3.4 ± 0.8	5.5 ± 1.6	< 0.001
E/A ratio	0.9 ± 0.3	0.9 ± 0.4	0.9 ± 0.3	0.645
E/e′ ratio	7.1 ± 2.5	7.0 ± 2.3	7.1 ± 2.5	0.883
PASP (mmHg)	32.3 ± 11.7	30.8 ± 11.3	32.7 ± 11.8	0.481
TAPSE (mm)	19.1 ± 4.9	14.8 ± 4.4	20.1 ± 4.4	< 0.001
GLS (%)	− 13.9 ± 3.6	− 9.3 ± 2.2	− 15.0 ± 3.0	< 0.001
TMAD 1 (mm)	10.9 ± 4.1	6.5 ± 2.5	11.9 ± 3.7	< 0.001
TMAD 2 (mm)	13.1 ± 5.3	7.6 ± 3.1	14.3 ± 4.9	< 0.001
TMADMid (mm)	11.5 ± 4.6	6.6 ± 2.6	12.6 ± 4.2	< 0.001
%TMAD	16.1 ± 6.3	9.5 ± 3.9	17.6 ± 5.8	< 0.001

LA, left atrium; LVEDV, left ventricle end-diastolic volume; LVESV, left ventricle end-systolic volume; LVEF, left ventricle ejection fraction; MAPSE, mitral annular plane systolic excursion; MA Smax, maximal lateral systolic mitral annular tissue velocity; E, early diastolic trans-mitral inflow velocity; A, late diastolic trans-mitral inflow velocity; e′, early lateral diastolic mitral annular tissue velocity; a′, late lateral diastolic mitral annular tissue velocity; LVOT VTI, left ventricular outflow tract velocity time integral; TAPSE, tricuspid annular plane systolic excursion; PASP, pulmonary arterial systolic pressure; CO, cardiac output; GLS, global longitudinal strain; TMAD1, septal tissue motion annular displacement; TMAD2, lateral tissue motion annular displacement; TMADMid, midpoint tissue motion annular displacement; and %TMAD, percentage value of the midpoint displacement in relation to the total length of the left ventricle

**Fig. 4:**
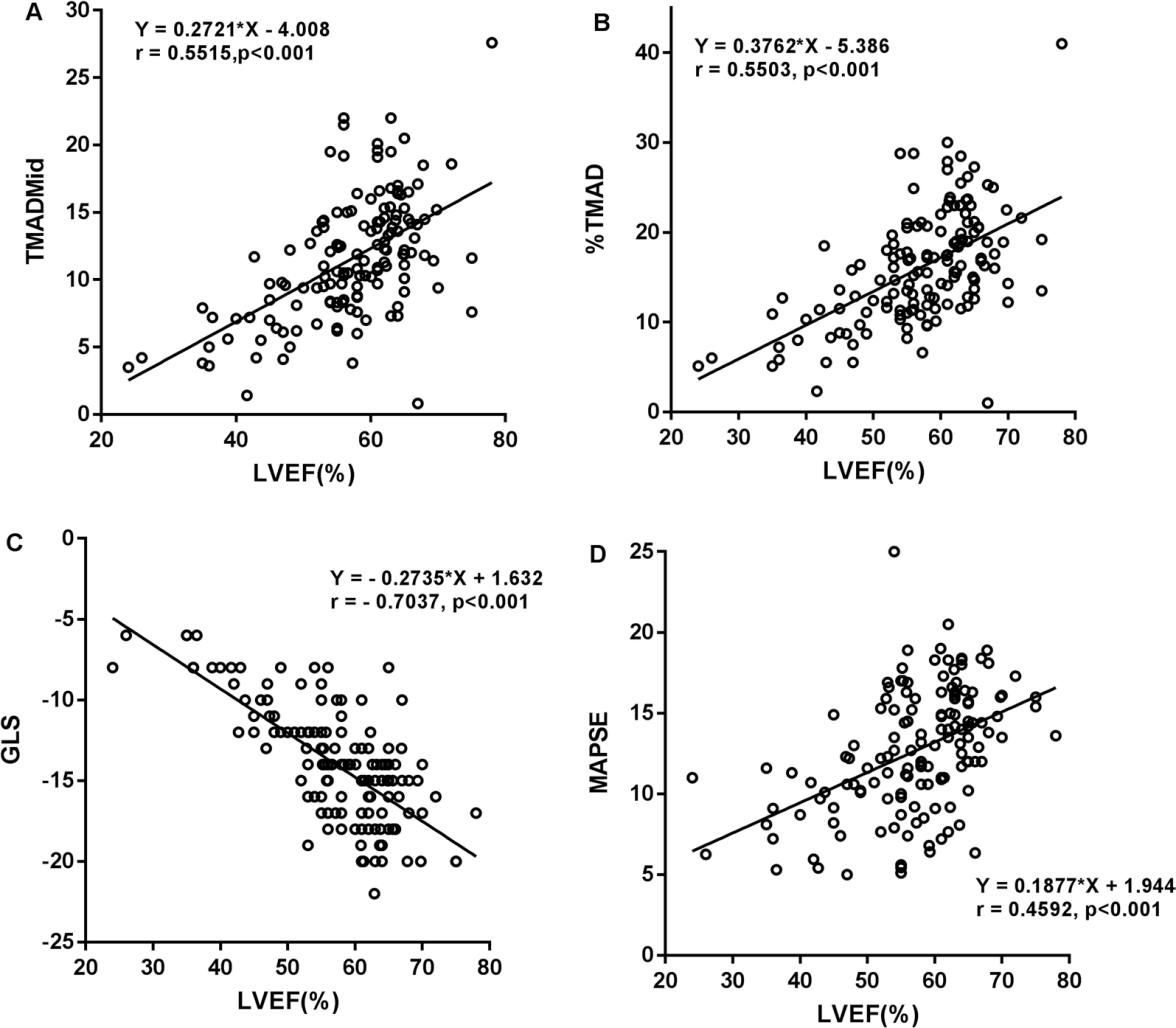
Relationship between LVEF and other echocardiographic parameters. **A** Positive correlation between LVEF and TMADMid. **B** Positive correlation between LVEF and %TMAD. **C** Negative correlation between LVEF and GLS. **D** Positive correlation between LVEF and MAPSE. TMADMid, midpoint tissue motion annular displacement; %TMAD, the percentage value of the midpoint displacement in relation to the total length of the left ventricle; LVEF, left ventricular ejection fraction; GLS, global longitudinal strain; and MAPSE, mitral annular plane systolic excursion

On the other hand, the correlations between TMAD, GLS, MAPSE and LVEF were analyzed. Positive correlations were detected between TMADMid, %TMAD, MAPSE and LVEF, respectively, while a negative correlation between GLS and LVEF was confirmed (all *p* < 0.001) (Fig. 4).

#### Discriminatory value of different echocardiographic parameters for SICM

**Fig. 5:**
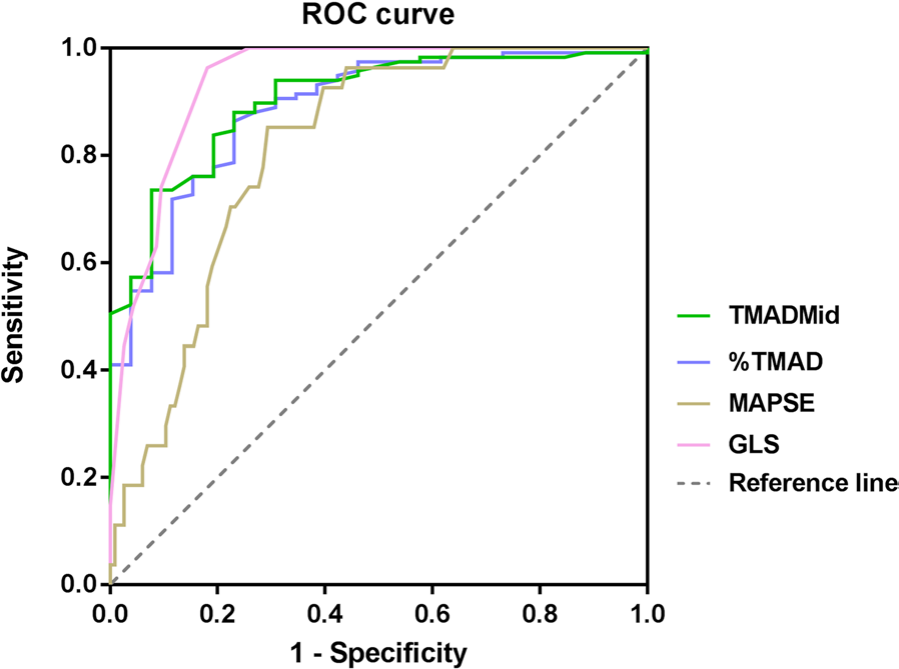
ROC curves to estimate the discriminatory value of TMAD for SICM compared with MAPSE and GLS. TMADMid, midpoint tissue motion annular displacement; %TMAD, the percentage value of the midpoint displacement in relation to the total length of the left ventricle; MAPSE, mitral annular plane systolic excursion; and GLS, global longitudinal strain

The discriminatory values of TMAD, GLS and MAPSE for predicting SICM defined as LVEF < 50% were evaluated. The AUROC values of TMADMid, %TMAD, GLS and MAPSE were 0.902, 0.887, 0.938 and 0.812, respectively (Fig. 5). The cutoff value, sensitivity and specificity of each parameter are listed in Table 4.

**Table 4:** AUROC curve analysis of TMADMid, %TMAD, GLS and MAPSE according to LVEF criteria

	AUROC	Cutoff value	95% CI	Sensitivity	Specificity	*p* value
TMADMid	0.902	9.75	0.844–0.961	73.5	92.3	*p* < 0.001
%TMAD	0.887	11.55	0.819–0.954	86.3	76.9	*p* < 0.001
GLS	0.938	− 12.5	0.900–0.975	96.3	81.9	*p* < 0.001
MAPSE	0.812	11.65	0.738–0.886	85.2	70.7	*p* < 0.001

TMADMid, midpoint tissue motion annular displacement; %TMAD, percentage value of the midpoint displacement in relation to the total length of the left ventricle; GLS, global longitudinal strain; and MAPSE, mitral annular plane systolic excursion

**Fig. 6:**
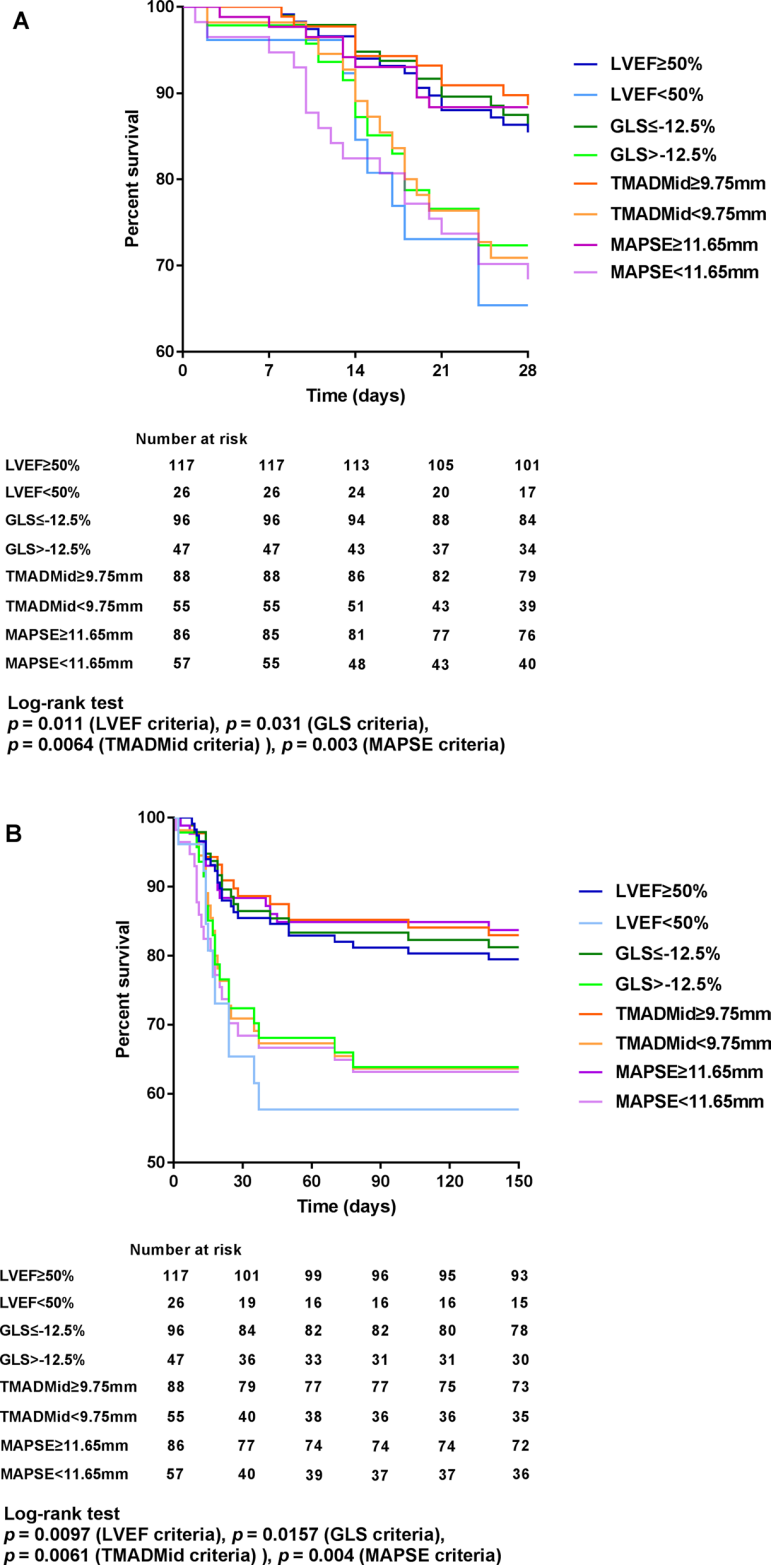
Kaplan–Meier survival curves according to the cutoff values of different echocardiographic parameters. **A** The 28d survival rates of SICM patients were significantly lower than that of non-SICM patients. **B** The in-hospital survival rates of SICM patients were significantly lower than that of non-SICM patients. SICM, sepsis-induced cardiomyopathy; LVEF, left ventricular ejection fraction; GLS, global longitudinal strain; TMADMid, midpoint tissue motion annular displacement; and MAPSE, mitral annular plane systolic excursion

Kaplan–Meier curves according to the cutoff values of each parameter were drawn (Fig. 6). The results showed that the 28d and in-hospital mortality were significantly higher in patients with TMADMid < 9.75 mm (29.1% vs. 10.2%; 36.4% vs. 17.0%), GLS > -12.5% (27.7% vs. 12.5%; 36.2% vs. 18.8%) or MAPSE < 11.65 mm (29.8% vs. 11.6%; 36.8% vs. 16.3%) (all *p* < 0.05).

### Prognostic value of TMAD for 28d and in-hospital mortality

Clinical and echocardiographic data of 28d and in-hospital survivors and non-survivors were collected and are shown in Table 5. TMADMid were significantly higher in both 28d and in-hospital survivors than non-survivors. However, cox proportional hazard model analysis showed that only BMI and SOFA were independent risk factors for both 28d and in-hospital mortality (Table 6).

**Table 5:** Clinical and echocardiographic data between survivors and non-survivors

	28d mortality			In-hospital mortality		
	Survivors	Non-survivors	*p* value	Survivors	Non-survivors	*p* value
Age (yrs)	66 ± 16	65 ± 21	0.624	66 ± 16	67 ± 19	0.887
Sex (male, %)	77 (65.3)	17 (68.0)	0.793	68 (63.0)	26 (74.3)	0.220
BMI (kg/m^2^)	23.2 ± 3.3	21.6 ± 2.9	0.027	23.3 ± 3.3	21.8 ± 2.8	0.021
APACHE II	16.3 ± 5.7	20.7 ± 7.2	0.001	15.8 ± 5.5	21.0 ± 6.7	< 0.001
SOFA	6.3 ± 2.4	9.1 ± 2.9	< 0.001	6.2 ± 2.4	8.6 ± 2.8	< 0.001
Comorbidity (*n*, %)	70 (59.3)	18 (72)	0.237	65 (60.2)	27 (77.1)	0.069
CVP (mmHg)	9.1 ± 2.8	10.6 ± 3.3	0.576	9.2 ± 2.9	10.7 ± 3.5	0.328
NT-proBNP (pg/mL)	3839 ± 610	8485 ± 2317	0.063	3368 ± 536	8609 ± 2000	0.016
CTnT (ng/mL)	0.07 ± 0.01	0.13 ± 0.03	0.048	0.06 ± 0.01	0.11 ± 0.03	0.059
CK-MB (ng/mL)	5.7 ± 1.0	10.3 ± 2.7	0.076	5.7 ± 1.0	8.8 ± 2.2	0.201
LVEF (%)	57.9 ± 8.6	53.3 ± 11.5	0.070	58.2 ± 8.5	53.8 ± 10.9	0.034
GLS (%)	− 14.1 ± 3.3	− 13.4 ± 4.7	0.475	− 14.2 ± 3.4	− 13.4 ± 4.2	0.322
MAPSE (mm)	12.9 ± 3.8	11.3 ± 3.5	0.053	13.1 ± 3.8	11.4 ± 3.5	0.022
TMADMid (mm)	12.1 ± 4.5	8.9 ± 4.1	0.001	12.2 ± 4.5	9.6 ± 4.2	0.003

BMI, body mass index; APACHE II, Acute Physiology and Chronic Health Evaluation II; SOFA, Sequential Organ Failure Assessment; CVP, central venous pressure; cTnT, cardiac troponin T; NT-proBNP, N-terminal pro-B type natriuretic peptide; CK-MB, creatine kinase-MB; GLS, global longitudinal strain; LVEF, left ventricle ejection fraction; MAPSE, mitral annular plane systolic excursion; and TMADMid, midpoint tissue motion annular displacement

**Table 6:** Predictors of 28d and in-hospital mortality using Cox proportional hazards model analysis

	28d mortality		In-hospital mortality	
	HR (95% CI)	*p* value	HR (95% CI)	*p* value
BMI (kg/m^2^)	0.718 (0.581–0.887)	0.002	0.874 (0.770–0.993)	0.038
APACHE II	1.051 (0.961–1.150)	0.274	1.000 (0.941–1.063)	0.997
SOFA	1.495 (1.181–1.894)	0.001	1.461 (1.217–1.753)	< 0.001
TMADMid (mm)	0.892 (0.784–1.016)	0.086	0.989 (0.898–1.089)	0.819
CTnT (ng/mL)	2.340 (0.047–116.702)	0.670	–	–
NT-proBNP (pg/mL)	–	–	1.000 (1.000–1.000)	0.901
LVEF (%)	–	–	1.024 (0.975–1.076)	0.341
MAPSE (mm)	–	–	0.900 (0.797–1.015)	0.086

BMI, body mass index; APACHE II, Acute Physiology and Chronic Health Evaluation II; SOFA, Sequential Organ Failure Assessment; cTnT, cardiac troponin T; NT-proBNP, N-terminal pro-B type natriuretic peptide; LVEF, left ventricle ejection fraction; MAPSE, mitral annular plane systolic excursion; and TMADMid, midpoint tissue motion annular displacement

## Discussion

In this study, we found that TMAD was feasible in 84.8% of eligible patients, which is higher than GLS and LVEF. TMAD also presented higher intra- and inter-observer reliability and less time consumed for measurement. Compared with MAPSE, TMAD had higher discriminatory value for predicting SICM. Patients with lower TMADMid (< 9.75 mm) had significantly higher 28d and in-hospital mortality. BMI and SOFA were the independent risk factors for 28d and in-hospital mortality.

Previous studies have demonstrated that LVEF decreased in patients with sepsis and the mortality of SICM patients was higher than those without SICM [26]. Paradoxically, patients with reversible decreases in EF had better outcomes than those without a decreased EF [27]. This may be explained that although LV systolic dysfunction should have resulted in a substantially decreased cardiac output, concurrent LV dilatation resulted in a preserved stroke volume, provided that fluid resuscitation was adequate. Therefore, studies of SICM were fraught with puzzling and contradictory findings. Despite uncertainties about its precise definition, we defined SICM as LVEF < 50% in this study which was reported in most previous literature [28]. According to the definition, the SICM incidence in our study was similar to most previous reported studies [2, 16, 29]. Consistent with some previous investigations [6, 7], SICM mortality in our study was significantly higher (about 2.5 times) than that of non-SICM patients.

As an indispensable hemodynamic diagnostic tool in the field of critical care medicine, echocardiography plays an important role in the evaluation of LV systolic and/or diastolic function, as well as in volume management. In recent years, there has been an increased interest in applying STE to evaluate the LV function in sepsis [15, 16, 30–32]. The strain imaging technique is based on regional myocardial deformation, and GLS, the mean longitudinal strain value from each segment of the LV [33], is the most frequently used strain parameter. In this regard, Chang et al. found that the LVEF was similar between survivors and deceased, while GLS values were significantly better in those survivors, with an even greater difference in ICU mortality [15]. Nevertheless, in clinical practice, we have indeed found that it is difficult to obtain clear echocardiographic images from critically ill patients. In this study, image acquisition rate of LVEF and GLS is significantly lower than that of MAPSE and TMAD. Poor continuity in endocardial imaging causes inaccuracy in the LVEF and GLS measurements, which is, in turn, manifested in poor intra- and inter-observer repeatability. As mentioned above, a large proportion of patients were excluded in our study due to the poor quality of the echocardiographic images, especially poor endocardium continuity.

MAPSE is another simple, rapid and reliable method to assess LV longitudinal function, which has been shown to have good correlation with LV longitudinal strain in critically ill patients [34, 35]. In our study, MAPSE showed significant difference between SICM and non-SICM group, and a MAPSE of < 11.65 mm indicated decreased LVEF (< 50%) with a sensitivity of 85.2% and a specificity of 70.7%. However, MAPSE has several limitations, including preload and angle dependence, inability to detect regional myocardial abnormalities and variation due to different cardiac size (especially in children). According to our results, MAPSE had the smallest AUROC value, as well as the smallest intra- and inter-observer reliabilities compared with TMAD and GLS, which might be relevant to the limitations mentioned above.

The STE-based TMAD is a novel method for assessing LV longitudinal systolic function [36] and a technology with several advantages. First and foremost, TMAD is less dependent on echocardiographic image quality. Compared with LVEF and GLS, the TMAD feasibility is higher, because its measurement process only requires locating the mitral annulus and the cardiac apex, instead of tracing the whole distinct endocardium border [19]. Therefore, less perfect echocardiographic image quality is acceptable. In this study, a considerable number of patients were excluded because of discontinuous endocardium, which entailed the inability to complete LVEF or GLS measurement. This deficiency can be effectively circumvented by the TMAD technology. Second, TMAD assessment is easy, quick and angle independent. According to the semi-automatically detection principle, TMAD measurement omits the whole endocardium tracing step, which allows a quick completion and has a good correlation with LVEF [18, 36, 37]. In our study, we confirmed that the required time for TMAD offline analyses was significantly shorter than that for LVEF or GLS. And in our opinion, the feature of angle independent is the main reason why TMAD is more accurate and reliable than MAPSE. Third, TMAD presents significant correlations with many conventional LV systolic function parameters. In both animal and human researches, TMAD has been significantly correlated with LVEF and GLS [38–40]. Consistent with previous literature, we detected significant correlations between TMAD and LVEF. According to the AUROC value, TMADMid presented an excellent discriminatory value in predicting LVEF < 50%. Fourth, the TMAD examination is highly repeatable. Buss et al. reported that TMAD has a relatively low inter- and intra-observer variability [18], an aspect confirmed in the present study. Here, both intra- and inter-observer ICCs of TMAD were considerably high, and superior to that of LVEF, GLS and MAPSE. These advantages support the feasibility of TMAD for the accurate assessment of the LV longitudinal systolic function in septic patients for daily clinical practice. In this study, BMI and SOFA were proved to be the independent risk factors of 28d and in-hospital mortality for patients with sepsis, which is consistent with previous studies [41]. Furthermore, Zaky et al. found that TMAD was significantly correlated with the LV diastolic function, which was not confirmed in our study [25].

In this work, four TMAD parameters were collected. Among them, TMADMid represented the midpoint displacement between TMAD1 and TMAD2, which better reflected the global systolic function of LV. Additionally, %TMAD represented the percentage value of TMADMid in relation to the total length of the LV and was supposed to reduce the bias caused by the different LV sizes in different individuals. According to the results, TMADMid and %TMAD exhibited good-to-excellent diagnostic accuracy for SICM, while TMADMid presented higher specificity and %TMAD presented higher sensitivity. We believe that TMAD had promising prospects for the diagnosis of SICM.

Several limitations in this study should be considered. First, it was a retrospective single-center study with a relatively limited sample size. Several patients were excluded due to poor echocardiographic image quality or incomplete echocardiographic data, which might affect the results. Second, the possible effect of the annulus calcification on TMAD measurement was not assessed. Gökdeniz et al. found that GLS is correlated with the presence and severity of mitral annulus calcification [41]. In our study, the degree of mitral annulus calcification was also not assessed systematically, which might influence TMAD values in some patients. However, according to the excluding criteria, no patients with valvular heart disease were included. Third, the time window for the echocardiography examination was defined within 24 h after admission. Indeed, a small number of critically ill patients were not at their sickest when admitted to our center and did not develop SICM until 24 h or even longer, so grouping bias might exist among these patients. Fourth, right ventricular STE was not performed, while SICM might present as right ventricular insufficiency. We reported here that TAPSE in the SICM group was significantly lower than in the non-SICM group; however, the right ventricular longitudinal strain and the TMAD values of the tricuspid annulus were not collected. Therefore, additional well-designed prospective studies with a larger sample size and a comprehensive STE that includes both ventricles are expected to validate the present results in the future.

## Conclusion

SICM is a common disease among septic patients and reports significantly high mortality. STE-based TMAD is a novel and feasible technique for discriminating SICM defined as LVEF < 50% in patients with sepsis.

## Supplementary Information

**Additional file 1**. TMAD measurement steps on the Qlab software. Open the Qlab software and select a clear echocardiographic image. Select TMAD package of the software and three ROIs: the septal (TMAD1) and lateral (TMAD1) mitral annulus, and the apex of the LV. The midpoint (TMADMid) of TMAD1 and TMAD2 is automatically detected. Then tracking will be automatically performed frame by frame and the results of TMAD1, TMAD2, TMADMid, and %TMAD are calculated (%TMAD).

## Data Availability

All data generated or analyzed during this study are included in this manuscript. The corresponding author may provide specified analyses or fully de-identified parts of the dataset upon reasonable request.

## Abbreviations

SICM: Sepsis-induced cardiomyopathy
GLS: Global longitudinal strain
LVEF: Left ventricle ejection fraction
STE: Speckle tracking echocardiography
TMAD1: Septal tissue motion annular displacement
TMAD2: Lateral tissue motion annular displacement
TMADMid: Midpoint tissue motion annular displacement
%TMAD: Percentage value of the midpoint displacement in relation to the total length of the left ventricle
BMI: Body mass index
APACHE II: Acute Physiology and Chronic Health Evaluation II
SOFA: Sequential Organ Failure Assessment
COPD: Chronic obstructive pulmonary disease
CCB: Calcium channel blocker
ACEI: Angiotensin-converting enzyme inhibitor
ARB: Angiotensin receptor blocker
HR: Heart rate
MAP: Mean arterial pressure
CVP: Central venous pressure
ICU: Intensive care unit
MV: Mechanical ventilation
CRRT: Continuous renal replacement therapy
ECMO: Extracorporeal membrane oxygenation
cTnT: Cardiac troponin T
NT-proBNP: N-terminal pro-B type natriuretic peptide
CK-MB: Creatine kinase-MB
LA: Left atrium
LVEDV: Left ventricle end-diastolic volume
LVESV: Left ventricle end-systolic volume
MAPSE: Mitral annular plane systolic excursion
TAPSE: Tricuspid annular plane systolic excursion
PASP: Pulmonary arterial systolic pressure
E: Early diastolic trans-mitral inflow velocity
A: Late diastolic trans-mitral inflow velocity
e′: Early lateral diastolic mitral annular tissue velocity
a′: Late lateral diastolic mitral annular tissue velocity
MA Smax: Maximal lateral systolic mitral annular tissue velocity
LVOT VTI: Left ventricular outflow tract velocity time integral
SV: Stroke volume
CO: Cardiac output
ICC: Intraclass correlation coefficient
